# Invasion and replication of *Yersinia ruckeri* in fish cell cultures

**DOI:** 10.1186/s12917-018-1408-1

**Published:** 2018-03-09

**Authors:** Simon Menanteau-Ledouble, Mark L. Lawrence, Mansour El-Matbouli

**Affiliations:** 1 Clinical Division of Fish Medicine, University of Veterinary Medicine, Veterinärplatz 1, 1210 Vienna, Austria; 2 Department of Basic Sciences, College of Veterinary Medicine, Mississippi State University, Mississippi State, MS USA

**Keywords:** Intracellular invasion, Gentamycin assay, Atlantic Salmon kidney cells, Chinook Salmon embryo cells, Salmon head kidney cells

## Abstract

**Background:**

Like many members of the *Enterobacteriaceae* family, *Yersinia ruckeri* has the ability to invade non professional phagocytic cells. Intracellular location is advantageous for the bacterium because it shields it from the immune system and can help it cross epithelial membranes and gain entry into the host. In the present manuscript, we report on our investigation regarding the mechanisms of *Y. ruckeri*’s invasion of host cells.

**Results:**

A gentamycin assay was applied to two isolates, belonging to both the biotype 1 (ATCC 29473) and biotype 2 (A7959–11) and using several cell culture types: Atlantic Salmon Kidney, Salmon Head Kidney and, Chinook salmon embryos cells at both low and high passage numbers. Varying degrees of sensitivity to *Y. ruckeri* infection were found between the cell types and the biotype 1 strain was found to be more invasive than the non-motile biotype 2 isolate. Furthermore, the effect of six chemical compounds (Cytochalasin D, TAE 226, vinblastine, genistein, colchicine and, N-acetylcysteine), known to interfere with bacterial invasion strategies, were investigated. All of these compounds had a significant impact on the ability of the bacterium to invade host cells. Changes in the concentration of bacterial cells over time were investigated and the results suggested that neither isolate could survive intracellularly for sustained periods.

**Conclusions:**

These results suggest that *Y. ruckeri* can gain entrance into host cells through several mechanisms, and might take advantage of both the actin and microtubule cytoskeletal systems.

## Background

*Yersinia ruckeri* [1] is a member of the *Enterobacteriaceae* family [2]. It is mostly known as a disease agent in salmonid fish even if many more species are susceptible to the disease [3]. Infections have been associated with a generalized bacteraemia and septicaemia as well as petechial and hemorrhagic lesions, particularly in the oral region [4]. While efficient bacterin-based vaccination has been introduced and has been widely adopted to control the bacterium [2], non-motile isolates belonging to the biotype 2 have been reported that are able to cause disease even in vaccinated fish [5, 6]. More recently, new vaccines have been made commercially available that contain both the biotype 1 and the biotype 2 and aim at protecting the fish against either biotypes of the bacterium [7].

Several *Enterobacteriaceae*, such as the fish bacterial pathogens *Edwardsiella ictaluri*, have demonstrated the ability to invade non professional phagocytic cells [8]. Not only can such intracellular location shield bacteria from the immune system [9], it can provide an environment richer in limited compounds such as iron.

*Y. ruckeri* was also shown to invade epithelial cells [10–12]; however, this ability has received limited scrutiny. Most notably, Tobback et al. [12] used a variety of techniques to demonstrate the ability of several isolates of *Y. ruckeri* to invade Chinook salmon embryo cell line (CHSE-214), fathead minnow epithelial cell line (FHM) and rainbow trout liver cell line (R1). The present study is a continuation of their work. In other members of the *Enterobacteriaceae* family, several strategies have been described that allow the bacterium to take advantage of host mechanisms to gain entry into the eukaryotic cells. One of these strategies is termed the “zipper mechanism” and has been described in *Yersinia enterocolitica* and *Yersinia pseudotuberculosis*. In these species, adhesin molecules on the surface of the bacteria interact with receptors on the surface of the host cell. For example, both *Yersinia* invasins [13] and adhesin A (YadA) [14, 15] proteins targets the β1-integrins. The interactions between these molecules initiate a signalling cascade that includes the signalling proteins Rac1 and CDC42 as key components. This signalling cascade results in the recruitment of additional receptors, in particular focal adhesion kinase (FAK), to the area [16, 17] and these additional receptors bind to the surface of the bacterium, folding the host cell’s membrane to engulf the bacterium [17].

The other main mechanism of entry described in *Enterobacteriaceae* is the trigger mechanism, which relies on the type III secretion system (T3SS) to inject effector proteins into the cytoplasm of the host cell where they activate various proteins belonging to the Rho family, including CDC42 and Rac1. These proteins regulate the activity of actin filaments and the formation of filopodial and lamellipodial structures that allow for cell migration as well as the cytoskeletal deformation required for phagocytosis. Effector proteins secreted through the T3SS can therefore trigger the uptake of the bacterial pathogen by the host cells [18].

An example of the trigger mechanism has been described for the Ssa-Esc family of T3SS. The Ssa-Esc family is one of the major families of T3SS, and it has been most studied in *Salmonella* and enterohaemorrhagic *Escherichia coli* [19]. About 30 effector proteins are known to be secreted through T3SSs belonging to the Ssa-Esc family, and five of these proteins (SopE/E2, SopB, SipA, SipC and SptP) play a role in the internalisation of *Salmonella* by non phagocytic cells. SopE/E2 and SopB activate Rho GTPase, which leads to the activation of Cdc42 and Rac1 [20], resulting to the remodelling of actin and the formation of filopodial structures. SipA and SipC induce polymerization and bundling of actin, and SopB causes the generation of macropinosomes and *Salmonella* containing vacuoles (SCVs). The role of SptP is to revert these changes and restore a normal state in the host cell [21, 22].

Interestingly, T3SSs belonging to the Ssa-Esc family have also been described in members of the *Yersinia* genus [23], although in *Yersinia pestis* several genes appear missing from the bacterial genome rendering this secretion system non-functional [24]. A T3SS belonging to the Ssa-Esc family has also been identified in several *Y. ruckeri* [25, 26], which suggests that the trigger mechanism could possibly play a role in the invasiveness of *Y. ruckeri*. However, very little is known about *Y. ruckeri*’s T3SS and its repertoire of effector proteins.

The present study was designed to investigate the targets of *Y. ruckeri*’s invasion mechanisms and expend on the work previously reported by Tobback et al. [12], in particular by confirming that their results applied to other cell lines beyond the ones they tested as well as by investigating if the non-motile biotype 2 isolate showed the same ability to invade host cells. Consequently, we measured the ability of two *Y. ruckeri* isolates, ATCC 29473 (biotype 1) and A7959–11 (biotype 2), to adhere to Chinook Salmon Embryo 214 (CHSE-214) cell cultures as well as to invade CHSE-214 cells at both a low and high passage number and Atlantic Salmon Kidney ATCC® CRL-2747 (ASK) and Salmon Head Kidney (SHK) cell cultures. Furthermore, the effects of several chemical compounds known to interfere with bacterial invasion strategies were also studied to improve our understanding of the host mechanisms targeted by *Y. ruckeri*. Finally, changes in the intracellular survival of bacteria over time were also investigated to assess the ability of the bacteria to survive intracellularly over prolonged periods of time.

## Methods

### Bacterial isolates and cell strains

Two *Y. ruckeri* isolates were used in this study: ATCC 29473 is a type strain and a motile isolate that belongs to the biotype 1. A7959–11 is a non-motile clinical isolate that originated from an outbreak in rainbow trout (*Oncorhynchus mykiss*) in Austria and belongs to the biotype 2. In our experience, the virulence of ATCC 29473 is much lower than that of A7959–11 in rainbow trout (*O. mykiss*), with LD50 by immersion of 10^10^ and < 6.5 × 10^5^, respectively.

To confirm that spectrophotometry was an accurate way to measure and normalize the concentration of both isolates, both *Y. ruckeri* ATCC 29473 and A7959–11 were cultivated overnight in brain heart infusion (BHI, Oxoid). Afterwards, the optical densities (OD) in the cultures were measured by spectrophotometry at 600 nm, and both cultures were diluted to the same OD of 0.5 at 600 nm. Both cultures were serially diluted, spread on BHI-agar, and incubated overnight at 22 °C. The number of colony forming units (CFU) on the plates were quantified and compared.

ASK and CHSE-214 cells originated from the American Type Culture Collection (ATCC) while SHK originated from the National Collection of Type Cultures Culture Collections subdivision from the Health Protection Agency (HPA). All cell lines were obtained through Sigma-Aldrich (Austria) except for ASK that was obtained directly from the ATCC.

### Invasion assay

Assays were conducted in 24 well plates following the procedure described by Skirpstunas and Baldwin [8] with some minor modifications: because of *Y. ruckeri*’s lower optimal temperature, all assays were performed at room temperature rather than 26 °C. Moreover, because of this lower temperature, the duration of the first incubation time was increased to five hours.

These assays were performed on ASK, SHK, and two strains of CHSE-214 cells with a different passage number: Earlier work in our laboratory had indicated that the older CHSE-214 cells showed different sensitivity to viral infection than the younger ones. Furthermore they adhered less strongly to the culture flasks suggesting that they might express surface adhesion molecules differently. Because of the important role that such adhesion molecules can play in the adherence and invasiveness of bacterial pathogens, including within the genus *Yersinia* [27, 28], it was decided to compare both old and young CHSE-214 strains.

Two cell types were used at a time: CHSE-214 cells with a low passage number were cultivated in the 12 leftmost wells of a 24 well plate while another cell type was cultivated in the remaining 12 wells. The cells were cultivated in minimum essential medium (MEM, Gibco) until they reached confluence. At the same time, both bacterial isolates were grown overnight in BHI at 20 °C. Their concentration was assessed by spectrometry at 600 nm, and the cultures were diluted in broth to the same optical density of 0.5. The bacteria were centrifuged at 4000 g for 10 min and resuspended in an equal volume of MEM before being diluted 10 times in MEM. The culture medium was replaced in each well with 1 ml of the bacterial suspension, and the bacteria were left to interact with the cells for five hours at room temperature. Afterwards, the solution was removed, the cells were washed twice with un-inoculated medium, and the supernatant was replaced with medium containing the antibiotic gentamycin (Sigma-Aldrich) at a concentration of 100 μg ml^− 1^. The antibiotic was left to act for four hours, after which the cells were washed twice with culture medium before replacing the medium with medium containing 1% of the detergent Triton-X (Sigma-Aldrich). The cells were incubated in the detergent for 10 min before being triturated with a micropipette, serially diluted from 10^− 1^ to 10^− 4^, and spread on brain heart infusion agar (BHI agar, Oxoid) in quadruplicate. Each assay was performed at least four times.

To confirm the efficacy of the gentamycin selection, both isolates were cultivated to the OD600 of 0.5. Both cultures were centrifuged at 4000 g for 10 min and resuspended in an equal volume of MEM before being diluted 10 times in gentamycin-containing MEM (100 μg ml^− 1^). After four hours of incubation, the bacteria were serially diluted and the 10^0^, 10^− 1^, 10^− 2^ and 10^− 3^ dilution were plated on LB agar plate and incubated overnight at 25 °C. The absence of any bacterial growth confirmed that the bacteria needed to invade and take shelter into eukaryotic cells to survive the gentamycin assay.

Conversely, an experiment was performed using CHSE-214 cells. The procedure was similar to the gentamycin assay except that no gentamycin was added to the medium during the second incubation. Because it was estimated that the bacterial count would be higher in the absence of gentamycin, serial dilution was performed up to 10^8^. This resulted in 5.62 × 10^8^ and 8.43 × 10^8^ CFU per ml for the ATCC 29473 and the A7959–11 isolates, respectively. No significant difference was found between these two isolates (*P* = 0.309), however a significant effect was found for the gentamycin (*P* < 0.05) for both cell types confirming that this antibiotic was highly efficacious at killing extracellular *Y. ruckeri*.

Moreover, for each assay, cells were left uninfected to use as a negative control to confirm that no bacteria could be recovered from the uninfected plates and confirm the absence of bacterial contamination.

In addition, another control assay was performed during which uninfected cells were exposed to Triton X and triturated in the manner previously mentioned. The cells were then stained with Trypan blue and observed using inverted microscopy to confirm that this procedure resulted in the lysis of the cultured cells.

Conversely, another control was performed in which the infection procedure was performed as usual save that no Triton-X was added at the end and not trituration took place. No bacteria could be recovered from the supernatant confirming that these steps were necessary to the release of the bacteria.

### Blocker assay

The next step of the experiment was designed to investigate the effectiveness of various chemical blockers to interfere with the bacterium’s ability to invade host cells. To simplify and streamline the investigation and because it was felt that the mechanisms of infection did not vary qualitatively between cell types and isolates, the research focussed on the CHSE-214 cell line with a low passage number and the *Y. ruckeri* isolate ATCC 29473. The bacteria were resuspended to an optical density of 0.5 nm in culture medium (MEM, Gibco) into which a working solution of one of the blockers was added (Table 1) and incubated alongside the cells for five hours. Afterwards, the cells were rinsed three times with PBS and the same working solutions were added to MEM supplemented with gentamycin (100 μg ml^− 1^). After four hours, the cells were lysed by 10 min incubation in medium supplemented with Triton-X (1%) followed by trituration before being serially diluted from 10^− 1^ to 10^− 4^, and spread on BHI agar. Each assay was performed five times.

**Table 1:** Effect of the chemical blockers on the intracellular invasion of *Yersinia ruckeri*

Blocker	Working concentration	Average CFU/ml	Standard deviation	Significance	Percentage inhibition
Control	–	3.9 × 10^4^	2.35 × 10^4^	–	–
Cytochalasin D	2 μM	4.8 × 10^2^	1.7 × 10^2^	*P* < 0.05	98.8%
TAE 226	10 μM	2.4 × 10^4^	1.6 × 10^4^	*P <* 0.05	39.0%
Vinblastine	10 μM	3.4 × 10^3^	1.1 × 10^3^	*P* < 0.05	91.5%
Genistein	1 mM	4.9 × 10^3^	2.2 × 10^3^	*P* < 0.05	87.5%
Colchicine	2 μM	1.7 × 10^3^	1.3 × 10^3^	*P* < 0.05	95.8%
N-acetylcysteine	5 mM	0.0	0.0	*P* < 0.05	100.0%

The numbers presented are based on *Y. ruckeri* strain ATCC 29473 infection a Chinook Salmon Embryo cell culture

Six blockers were investigated: Cytochalasin D, TAE 226, vinblastine, genistein, colchicine and, N-acetylcysteine (all blockers were obtained from Sigma Aldrich except TAE 226, which was acquired from Apexbio technology). Each blocker was assayed in quadruplicate.

To investigate any potential toxic effect of the blockers on the survival of both bacteria and host cells, control tests were performed in which bacteria were incubated alongside the blockers for five hours before being serially diluted and plated. Another control exposed the cell cultures to the blockers. Afterwards, a solution of Trypan Blue was added to the cells and incubated for one minute. The cells were then fixed by addition of formalin to a concentration of 4%. After ten minutes, the formalin was removed and the cells were rinsed 3 times in PBS. The cells were then observed using an inverted microscope. Each cell types was observed five times and each time a hundred cells were counted and the number of blue cells counted in order to obtain the percentage of live versus dead cells. The results showed that N-acetylcysteine had a negative impact on the survival of the host cells (*P* = 0.02).

### Adhesion assay

An adhesion assay was performed based on a modified version of the protocol designed by Kotzamanidis et al. [29]: Bacterial cells belonging to both the isolate A7959–11 and ATCC 29473 were used in parallel. The bacterial cultures were grown overnight and their concentration was adjusted to an O.D of 0.5. After that, they were added to the CHSE-214 cell cultures as previously described. Because this section of the experiment aimed at investigating the initial adhesion on the cells and not the bacterial multiplication, a shorter incubation time of 90 min at room temperature was chosen. Afterwards, the cells were fixed by addition of formalin to a concentration of 4%. The medium was removed and the cells were washed three times with PBS to rinse away the bacterial cells that were not attached. The cells and bacteria were stained using Giemsa before being imaged by inverted microscopy (Leica DM IRB). 100 CHSE-214 cells were observed and the number of attached bacteria was counted. This was performed five times for both isolates to determine the number of attached bacteria per hundred cells. Based on Kotzamanidis et al. [29], it was determined that the bacteria were strongly adhesive if more than 40 bacterial cells were found per hundred CHSE-214 cells.

### Intracellular replication assay

To assess the ability of the bacterium to survive and multiply inside host cells, another gentamycin assay was performed using CHSE-214 cells and both *Y. ruckeri* isolates.

In this assay too, incubation times varied in order to capture the change in bacterial concentration overtime: the bacteria were left to interact with the cells for one hour, after which the medium was replaced with one containing gentamycin (100 μg ml^− 1^). The antibiotic was left to interact with the cells for two, six, or fifteen hours (for a total infection time of three, seven, and sixteen hours). At the end of the incubation period, the blocker containing medium was removed, and the cultures were rinsed twice before being lysed with Triton X and plated. Each combination of isolate and time point was assayed in triplicate.

Moreover, the same protocol was repeated a second time with the exception that MEM supplemented with 10% formalin was added at the end of each incubation period rather than MEM supplemented with Triton X. The cells were incubated with formalin for 10 min, afterwards the medium was removed and the cells were washed twice with phosphate buffered saline (PBS). Once all the stain had been fixed a solution of GIEMSA was added on top of the cells, left to incubate for 5 min then rinsed three times in PBS. Afterwards, the cell cultures were examined using an inverted microscope to assess damage to the cell tissue visually.

### Statistical analysis

The number of colony forming units was determined on each plate, and the corresponding number of surviving bacteria per ml was calculated. The distributions of these numbers were checked for normality using Levene’s test. When the distribution was normal, analysis of variance was performed. Because normality could not be assumed in many cases, a non-parametric test was then performed using a Dunnet’s T-test. All statistics were performed using SPSS 24 (IBM).

## Results

### Susceptibility of the various strains and invasiveness of the two isolates

Initial serial dilution showed comparable bacterial concentrations between both isolates cultivated at an identical OD of 0.5 at 600 nm: 1.5 × 10^09^ CFU/ml for *Y. ruckeri* ATCC 29473 and 8.4 × 10^08^ CFU/ml for *Y. ruckeri* A7959–11 (*P* = 0.748).

**Fig. 1:**
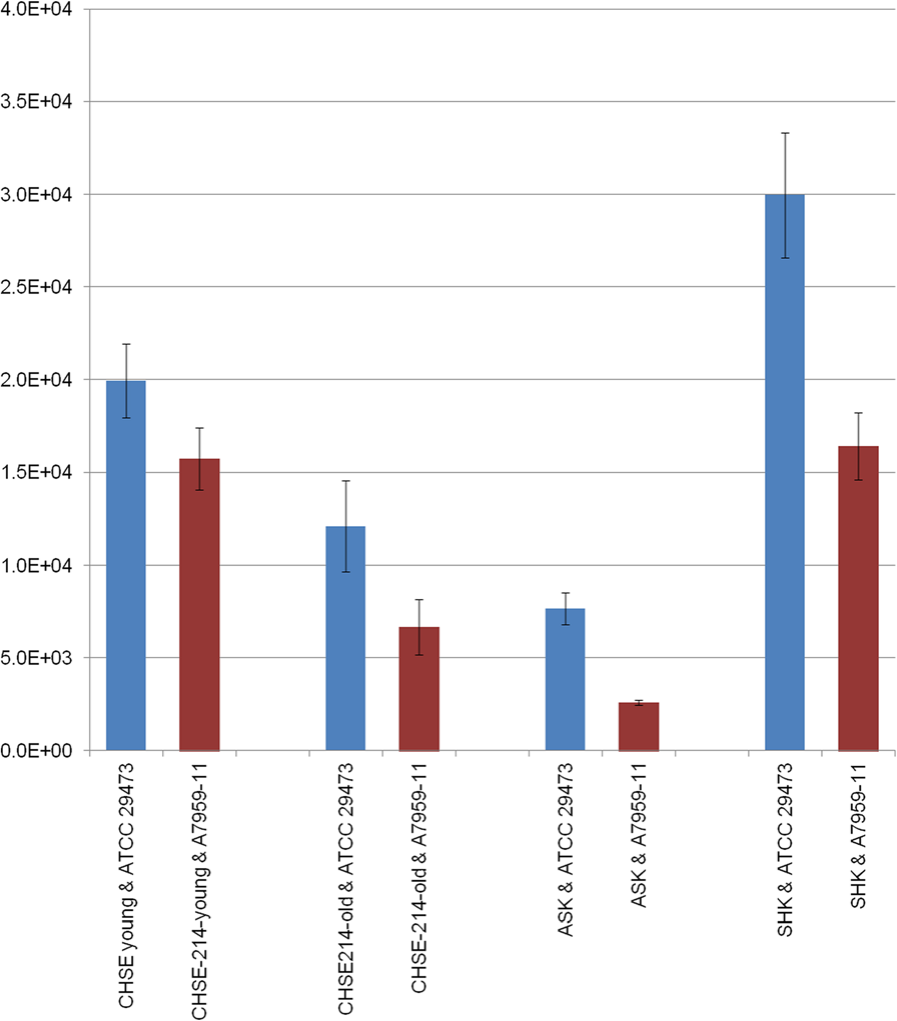
Susceptibility of the various cell types tested to ATCC 29473 and A7959–11. The number represents the average number of attached bacterial cells per ml. Error bars represent the standard errors values

CHSE-214 cells with a low passage number were more susceptible than both CHSE-214 cells with a high passage number (Fig. 1) and ASK (*P* < 0.05) but not significantly different compared to SHK (*P* = 0.44). Similarly, SHK was significantly more susceptible than all cell type (*P* < 0.05) with the exception of CHSE-214 with a low passage number.

Overall, the ATCC 29473 strain was significantly more infectious than isolate A7959–11 in most cell types: The average number of surviving bacteria across all cell types was 1.89 × 10^4^ CFU/ml for the ATCC 29473 strain and 1.38 × 10^4^ CFU/ml for the isolate A7959–11 (*P* < 0.05). However, this difference was not as strong when only considering the CHSE-214 cells: while more *Y. ruckeri* of the ATCC 29473 strain were recovered (1.6 × 10^4^ CFU/ml compared to 1.1 × 10^4^ CFU/ml), this difference was not significant (*P* = 0.133).

### Blocker activity

**Fig. 2:**
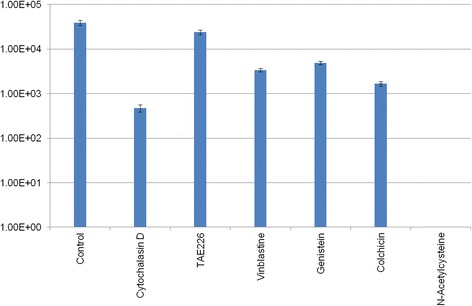
Effect of the chemical blockers on the intracellular invasion of *Yersinia ruckeri* ATCC 29473 on CHSE-214 cells. Error bars represent the standard errors values

In all cases, addition of the chemical blockers was associated with a significantly reduced bacterial concentrations (Fig. 2; *P <* 0.05), indicating that the blockers interfered with bacterial invasion. The blocker that had the least impact was TAE226, a compound that interferes with the activation of FAK on the surface of the host cells. The concentration of bacteria isolated at the end of the gentamycin assay was found to be 2.4 × 10^4^ CFU/ml, compared to 3.9 × 10^4^ CFU/ml in the untreated control cultures (*P* = 0.004). On the other hand, the blocker that had the most impact was the Rac1 inhibitor N-acetylcysteine, which totally blocked bacterial invasion (no bacteria were isolated) (Fig. 2, *P < 0.001*).

The controls showed no toxic effect of the blockers on the bacteria (*P* = 0.577). Interestingly, while most of the blockers did not affect the viability of the cells (*P* = 0.304), N-acetylcysteine did reduce the viability of the cells from 95.8% to 88.4% in the controls and N-acetylcysteine treated cells, respectively (*P* = 0.02).

### Intracellular replication assay

**Fig. 3:**
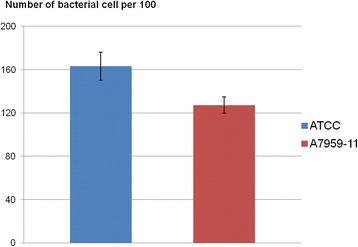
Adhesion of both isolates ATCC 29473 and A7959–11 during the adhesion assay. The number represents the average number of attached bacterial cells per hundred CHSE-214 cells after 90 min incubation at room temperature. Error bars represent the standard errors values

Both isolate were found to be strongly adhesive, based on the criterion by Kotzamanidis et al. [29]: 163 bacterial cells were found attached per hundred CHSE-214 cells for isolate ATCC 29473 and 127 were found for isolate A7959–11 (Fig. 3). Moreover, isolate ATCC 29473 was found to be significantly more adhesive than isolate A7959–11 (*P* = 0.044).

### Intracellular replication assay

**Fig. 4:**
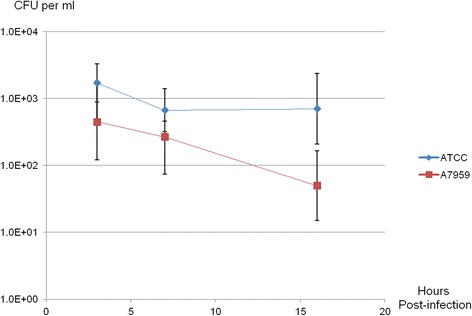
Concentrations from both isolate ATCC 29473 and A7959–11 at several time-points post-inoculation. The concentrations represent the mean bacterial numbers in CFU per ml at 1, 7 and 16 h during the gentamycin assay. Error bars represent standard deviations

For both isolates, the amount of bacteria surviving intracellularly decreased as the incubation time increased (Fig. 4). This was most evident for the biotype 2 isolate (A7959–11) where the number of bacteria was reduced from an average of 0.4 × 10^3^ CFU/ml after 3 h of incubation to 0.2 × 10^3^ CFU/ml after 7 h (*P* = 0.024) and 0.05 × 10^3^ CFU/ml after 16 h. Conversely, while the number of CFUs in the biotype 1 isolate (ATCC 29473) decreased between 3 and 7 h post-infection, from 1.7 × 10^3^ CFU/ml to 0.7 × 10^3^ CFU/ml (*P* = 0.036), this number appeared to stabilize after that point and did not differ significantly at 16 h (0.7 × 10^3^ CFU/ml; *P =* 0.935).

**Fig. 5:**
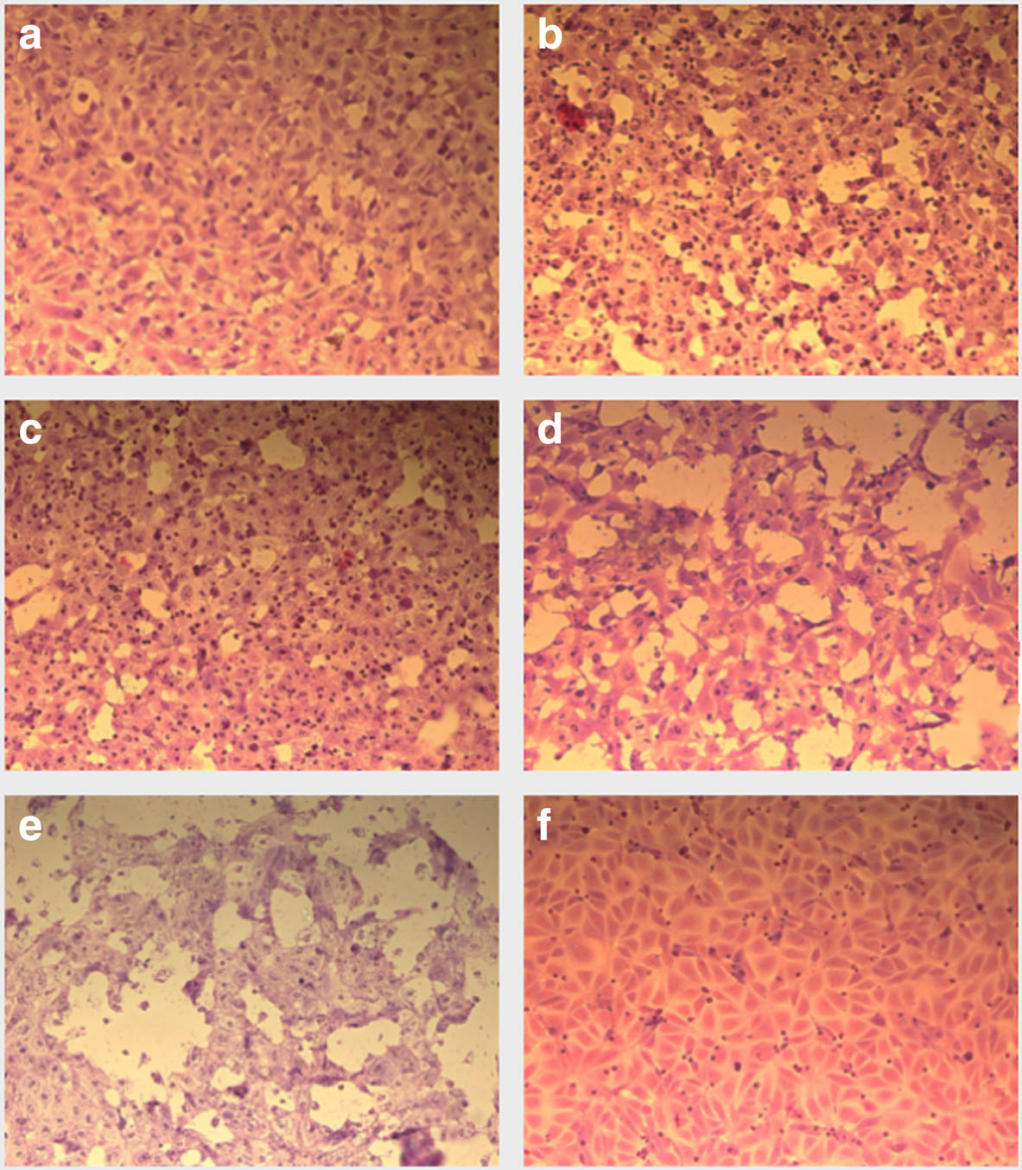
Inverted microscopy of cell layers infected with isolates ATCC 29473 and A7959–11 at several time-points post-inoculation. **a** Isolate ATCC 29470, 1 h post-infection; **b** Isolate A7959–11, 1 h post-infection; **c** Isolate ATCC 29470, 6 h post-infection; **d** Isolate A7959–11, 6 h post-infection; **e** Isolate ATCC 29470, 12 h post-infection; **f** Uninfected control, s post-infection

Upon microscopic examination, the tissue culture showed progressive deterioration as the bacteria damaged the cells. Tears appeared in the monolayer and cells became detached within 1 h post infection (Fig. 5). These changes were more marked at 6 h post infection, in particular in the cells infected with the A7959–11 isolate. By the 16 h time-point, most of the cell monolayer had been destroyed.

## Discussion

The various cell types displayed differing sensitivity to bacterial infection. This has been previously reported for several intracellular bacterial pathogens: for example, Elkamel and Thune [30] demonstrated than the number of *Photobacterium damselae* subsp. *piscicida* to survive a gentamycin assay was ten time higher in Fathead minnow (FHM) than in channel catfish ovary (CCO), or *Epithelioma papillosum cyprini* (EPC) cell cultures. Similarly, Skirpstunas and Baldwin [8] investigated the propensity of intestinal epithelial cells-6 (IEC-6), Henle 407, FHM, and trypsin/pepsin-harvested channel catfish enteric epithelial cells to invasion by *E. ictaluri* isolate LA89–9 and reported that the invasion efficiencies was lower in IEC-6 and Henle 407 than in FHM cells. Furthermore, the invasion efficiency was even lower in the trypsin/pepsin-harvested channel catfish cells, possibly because the harvesting process had damaged some surface receptors [8]. Finally, Tobback et al. investigated the ability of three *Y. ruckeri* isolate to invade several cell lines (CHSE-214, FHM and R1) [12]. These authors reported difference between the susceptibility of the various cell lines with CHSE-214 being the most susceptible. This is similar to our present results were both isolates were able to infect multiple cell line and where CHSE-214 was very susceptible to both isolates. In the present study we analysed two additional cell types beside CHSE-214 and confirmed that *Y. ruckeri* could also infect them. This appears to confirm that *Y. ruckeri* can infect a wide range of cell lines and that the mechanisms it targets as part of the invasion process are well conserved.

Interestingly, we observed a significant difference in sensitivity between CHSE-214 cell cultures with different passage numbers (*P* < 0.05). Effects of passage number on CHSE-214 phenotype had previously been noted in our laboratory with the younger cell CHSE-214 adhering more tightly to the culture flask than CHSE-214 with a higher passage number. Moreover, susceptibility to viral infection also varied between both cultures. This prompted determination of the effect of passage number in our study as adhesion molecules often play an important role as targets for entry of several members of the *Yersinia* genus [14–17]. Our findings suggest that adhesion molecules on the surface of the host cells might be targeted by *Y. ruckeri* invasion mechanisms. This would be consistent with *Y. ruckeri*’s ability to infect a wide-range of cells as adhesion molecules are well conserved [31]. Further studies will be required to confirm these results and identify the specific molecules targeted on the host cell surface.

ATCC 29473 was significantly more infectious than the non-motile isolate A7959–11 (*P* < 0.05), despite the latter originating from a clinical case and ATCC 29473 being less virulent in vivo in *O. mykiss*. Similarly, the type strain ATCC 29473 was significantly more infectious in most cell lines. Interestingly, it has been shown in *Y. enterocolitica* that motility contributed to bringing the bacteria in contact with the cells [32] and that non-motile isolates are less able to invade Human epithelial type 2 (HEp-2) cells. Based on the present findings, it is therefore possible that a similar situation exists in *Y. ruckeri*. This is consistent with findings reported by Kim in 2000 that suggested that the flagellum of *Y. ruckeri* plays a critical role in the early stages the bacterium’s attachment and entry into the host (Kim 2000, as quoted by Barnes in 2011 [2]).

We tested several chemical compounds that are known to interfere with aspects of the host cell’s physiology and hinder bacterial invasion. Because these various chemical compounds target different aspects of this physiology, comparing their effectiveness at hindering bacterial invasion can highlight which of the host’s mechanisms are taken advantage of by the bacterium.

Cytochalasin D, a mycotoxin interferes with actin filaments [33], prevents actin polymerization [34, 35] and inhibits membrane ruffling and blebbing [33]. In the present study, cytochalasin D reduced the ability of *Y. ruckeri* isolate ATCC 29473 to survive the gentamycin assay almost 100 fold (*P* < 0.05). This is similar with the results previously reported by Tobback et al. [12]. Colchicine hinders the microtubule cytoskeletal network as it depolymerises and destabilizes microtubules [36, 37]. Colchicine has previously been shown to hinder the invasion of Caco-2 (colonic carcinoma) cell lines by *Listeria monocytogenes* [36] as well as the invasion of HeLa by *Pseudomonas aeruginosa* [37]. Similar results were previously reported in *Y. ruckeri* by Tobback et al. [12] as well as in the present experiment (Table 1; Fig. 2.). TAE226 inhibits the extracellular matrix-dependent phosphorylation of the FAK integrin [38]. FAK is the target of *Campylobacter jejuni*’s fibronectin binding protein [38, 39]. In the present study, TAE226 appeared to have the least effect on the invasiveness of *Y. ruckeri* (*P* < 0.05; inhibition rate of 39.0%). This suggests that, while FAK might be involved in *Y. ruckeri*’s invasion of the host cells, this is not the only protein targeted by the bacterium. Vinblastine has the ability to react with microtubules, crystallizing them into helical structures [40] and addition of vinblastine has been shown to inhibit the invasion of intestine-derived epithelial cells (INT407) by *Enterobacter sakazakii* [41]. Here, vinblastine similarly hindered *Y. ruckeri*’s invasion of CHSE-214 (*P* < 0.05). Genistein blocks the binding of ATP to tyrosine phosphate kinase [42] and interferes with the phosphorylation of p38, p42/44, and, p-JNK, preventing polymerization of tubulin [43, 44]. Previously, genistein has been shown to strongly inhibit the invasion of HeLA cells by *E. coli* expressing the invasin protein cloned from *Y. enterocolitica* [9, 45]. In the present study, our results showed that genistein could also hinder invasion of *Y. ruckeri* into CHSE-214 cells. Interestingly, the inhibition rate in our experiment was lower than the one reported by Rosenshine et al. [9]: 87.5% compared to 98.5%. Because Rosenshine et al. [9] cloned one specific invasion molecule from *Y. enterolitica*; the lower inhibition rate observed in the present study could possibly be explained by *Y. ruckeri* having multiple redundant mechanisms to invade host cells. Finally, N-acetylcysteine has been shown to inhibit the activation of Rac1 by *Fusobacterium nucleatum* [46]. It plays as a regulator of both the actin [47, 48] and the microtubule cytoskeleton. Rac1 regulates the formation of lamellipodia at the edge of fibroblasts [49–51] and members of the *Enterobacteriacea* can activate Rac1 using proteins secreted via the T3SS, triggering membrane ruffling and phagocytic independent internalization of the bacterium [52]. In the present study, addition of N-acetylcysteine to a final concentration of 5 mM appeared to totally inhibit the ability of *Y. ruckeri* to invade cell cultures (*P* < 0.05). Controls have shown that N-acetylcysteine had a significant toxic effect on CHSE cells. This most likely contributed to its disruptive effect; however, it seems unlikely that the comparatively limited toxic effect accounts for the entirety of the invasion inhibition. Despite these chemicals affecting several different aspects of the host physiology, all blockers were efficacious at hindering *Y. ruckeri*’s invasion. Notably, some of the chemicals affect the actin filaments, and others inhibit the microtubule network. These results are consistent with that previously reported by Tobback et al. [12], however, additional blockers were tested in the present study alongside Cytochalasin D and colchicine. Our present results suggest that *Y. ruckeri* targets multiple host mechanisms to gain intracellular entry. This would be similar to what has already been described for other members of the genus *Yersinia*. The fact that N-acetylcysteine was the most efficacious of the chemical compounds tested would be consistent with this hypothesis as both the actin and microtubule cytoskeleton apparatus are regulated by Rac1, the target of N-acetylcysteine [53].

Bacterial survival decreased as the duration of the infection period increased (Fig. 3), again, this is consistent to what has been reported by Tobback et al. [12]. In the present study, we found that intracellular invasion destroyed the infected cells which would explain their inability to shield the bacteria for a sustained duration. Ohtani et al. [11] have shown that *Y. ruckeri* was able to invade the gill epithelial cells intracellularly and have suggested that the gill epithelium might constitute the bacterium’s main route of entry into the host. Our present findings are compatible with this proposition because sustained intracellular survival would not be required to cross-the epithelium. Indeed, Ohtani et al. [11] reported presence of *Y. ruckeri* in the blood as early as one minute after infecting the fish by immersion.

## Conclusion

In the present study, we build up on the experiments by Tobback et al. [12] and applied a series of gentamycin assays to investigate the ability of two isolates of *Y. ruckeri* to invade epithelial cells and found that all four of the cell types investigated (ASK, SHK and CHSE-214 with either a low or a high passage number) were susceptible to invasion. Furthermore the biotype 1 isolate (ATCC 29473) was significantly more infectious than our non-motile biotype 2 isolate (A7959–11), despite the fact that A7959–11 originated from a clinical case and was more virulent in vivo. This is reminiscent of what has been previously reported in *Y. enterocolitica* where non-motile isolates appeared less capable of intracellular invasion.

Chemical blockers targeting either the actin or microtubule cytoskeletal apparatus all reduced the susceptibility of CHSE-214 cells to invasion by the isolate ATCC 29473. This suggests that *Y. ruckeri* is able to initiate intracellular uptake through several host mechanisms. This hypothesis would be consistent with the fact that N-acetylcysteine, a compound that targets the regulator Rac1, appeared to completely prevent intracellular invasion as Rac1 is involved with the regulation of both the actin and microtubules cytoskeletons. Finally, it was shown that increasing the incubation time reduced bacterial survival, indicating that the bacterium is unable to survive intracellularly for long periods of time. This finding is likely explained by the bacteria quickly destroying the infected cells and would be consistent with the hypothesis that intracellular invasion plays a very specific role in the development of disease, for example as a mean of crossing the epithelium and gain entry into the host. To conclude, the present study confirms the ability of *Y. ruckeri* to facultatively invade a variety of host-cell lines. Interestingly, a difference was found between isolates belonging to the two biotypes.

## Abbreviations

ASK: Atlantic Salmon Kidney
ATCC: American Type Culture Collection
BHI: Brain Heart Infusion
CCO: Channel Catfish Ovary
CDC42: Cell division control protein 42 homolog
CFU: Colony Forming Units
CHSE-214: Chinook salmon embryos cells
EPC: Epithelioma papillosum cyprini
FAK: Focal Adhesion Kinase
FHM: Fathead minnow
GTP: Guanosine triphosphate
HeLA cells: Henrietta Lacks cell culture
Hep-2: Human epithelial type 2
HOK-16B: Human Oral Keratinocytes-16B
HPA: Health Protection Agency
MAPK: Mitogen Activated Protein Kinase
MEM: Minimum Essential Medium
PBS: Phosphate Buffered Saline
